# The Increase in the Plasticity of Microcrystalline Cellulose Spheres’ When Loaded with a Plasticizer

**DOI:** 10.3390/pharmaceutics16070945

**Published:** 2024-07-16

**Authors:** Artūrs Paulausks, Tetiana Kolisnyk, Valentyn Mohylyuk

**Affiliations:** 1 Leading Research Group, Faculty of Pharmacy, Riga Stradiņš University, 21 Konsula Str., LV-1007 Riga, Latvia; 2 School of Pharmacy, Queen’s University Belfast, 97 Lisburn Road, Belfast BT9 7BL, Northern Ireland, UK; 3 Department of Industrial Technology of Drugs, National University of Pharmacy, 53 Pushkins’ka Str., 61002 Kharkiv, Ukraine

**Keywords:** tablets, microcrystalline cellulose, plasticizer, glycerol, yield pressure, mean yield pressure, solubility parameters

## Abstract

Compaction pressure can induce an undesirable solid-state polymorphic transition in drugs, fragmentation, loss of coated pellet integrity, and the decreased viability and vitality of microorganisms. Thus, the excipients with increased plasticity can be considered as an option to decrease the undesirable effects of compaction pressure. This study aims to increase the plasticity (to reduce the mean yield pressure; *Py*) of dried microcrystalline cellulose (MCC) by loading it with a specially selected plasticizer. Diethyl citrate (DEC), water, and glycerol were the considered plasticizers. Computation of solubility parameters was used to predict the miscibility of MCC with plasticizers (possible plasticization effect). Plasticizer-loaded MCC spheres with 5.0 wt.% of water, 5.2 wt.% of DEC, and 4.2 wt.% glycerol were obtained via the solvent method, followed by solvent evaporation. Plasticizer-loaded formulations were characterised by TGA, DSC, pXRD, FTIR, pressure-displacement profiles, and in-die Heckel plots. *Py* was derived from the in-die Heckel analysis and was used as a plasticity parameter. In comparison with non-plasticized MCC (*Py* = 136.5 MPa), the plasticity of plasticizer-loaded formulations increased (and *Py* decreased) from DEC (124.7 MPa) to water (106.6 MPa) and glycerol (99.9 MPa), and that was in full accordance with the predicted miscibility likeliness order based on solubility parameters. Therefore, water and glycerol were able to decrease the *Py* of non-plasticized MCC spheres by 16.3 and 30.0%, respectively. This feasibility study showed the possibility of modifying the plasticity of MCC by loading it with a specially selected plasticizer.

## 1. Introduction

Being non-invasive and, in most cases, not requiring medical assistance, tablets for oral administration are the most widespread and the most popular pharmaceutical and nutraceutical dosage forms. Despite the rising topic of individualised/personalised medicine, including individualised dosing, drug release, and customer properties, national healthcare systems worldwide are highly dependent on the mass-market production of tablets and their usage following treatment protocols.

In the vast majority of cases, pharmaceutical substances cannot be converted into tablets via tableting with high-speed rotary tablet presses [1]. To achieve the desirable mechanical and biopharmaceutical properties, specific excipients are required. Appropriate mechanical properties, such as tablet hardness (or tensile strength) and abrasion resistance (friability) should ensure tablet applicability to transportation, coating, and packaging processes without losing their appearance, dose, and biopharmaceutical properties. Moreover, the intrinsic properties of tablet excipients and the structural-mechanical properties of the tablets formed eventually affect the disintegration and drug release behaviour of the dosage form, and so, can be deliberately selected to achieve the desired release profile [2].

**Figure 1:**
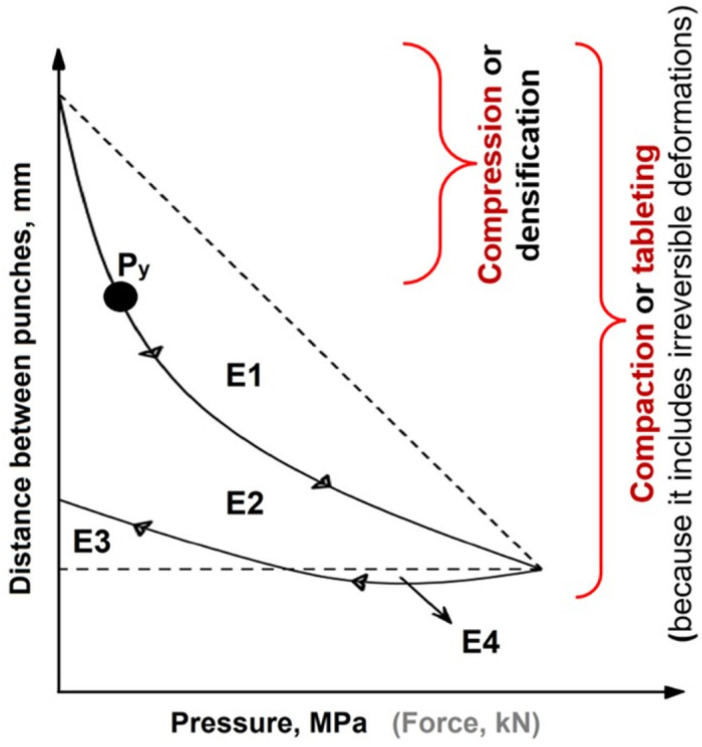
An example of a force-displacement profile highlighting: the rearrangement energy (E1), plastic energy (E2 + E4), elastic energy (E3; or energy lost), plastic flow energy (E4), compaction energy (E1 + E2 + E3), and mean yield pressure (*Py*). The arrows on the curve are showing the direction of curve development.

Upon tableting, the compaction pressure and tableting speed (dwell time) induce elastic and plastic deformation, or fragmentation, and affect the extent of these deformations [3]. The tableting cycle can be described with a force–displacement profile: the distance between punches, which is plotted against the compaction pressure or force. This can be determined with state-of-the-art equipment, such as compaction simulators containing hi-tech sensors and sophisticated user-friendly software [4,5] (Figure 1).

Considering the true density of the material, an in-die Heckel plot can be built: ln(1/porosity) is plotted against the compaction pressure. The greater the slope of the linear region (*K*), the greater the degree of plasticity of the material [6]. The mean yield pressure (*P_y_*) of the solid is reciprocal to *K* [7] and describes the point after which the deformation is irreversible (pointed out in Figure 1). It should be stressed that the mean yield pressure from the in-die Heckel analysis can be used as a reliable plasticity parameter: the lower the *P_y_*, the greater the degree of plasticity of the material [8].

Possessing information about the *Py* of each ingredient in the blend allows predicting the sequence of the events of the material irreversible deformations upon tableting cycle. Consequently, the targeted composition of a tableting blend based on excipients’ *Py* can predetermine the deformation (the extent of deformation) of the specific ingredients in this blend upon tableting at a specific compaction pressure [9]. Considering the possibility of undesirable solid-state polymorphic transition of the drug [10,11], particle fragmentation, the loss of coated pellet integrity [12,13], and the decreased viability and vitality of microorganisms [14,15] as a function of compaction pressure, the above-mentioned circumstances are of particular interest.

Microcrystalline cellulose (MCC) is a partially depolymerised, naturally occurring polymer in the form of crystalline powder or spheroids composed of porous particles [16], and it is one of the most commonly used excipients in tablet formulations [17]. MCC is used for direct compression (up to 90 wt.%), dried granulation/roll-compaction, and wet granulation to achieve tablets with desirable mechanical and biopharmaceutical properties [3,10,16]. MCC is recognised as an excipient with relatively low *Py* that undergoes plastic deformation at relatively low compression forces [3].

The effect of water on the plasticization of the MCC as well as its effect on the compaction properties upon tableting has been previously reported [18,19,20]. To the best of our knowledge, the information regarding MCC plasticization with other solvents or excipients in order to influence the compaction properties upon tableting is lacking. Nevertheless, the practice of modulating cellulose derivatives plasticity for film forming [21,22], hot-melt extrusion, and/or fusion deposition modelling 3D-printing [23,24] is common practice. While solubility parameters were found to be a useful instrument for plasticizer pre-screening [24,25].

This study aims to increase the plasticity (to reduce the *Py*) of MCC by loading it with a specially selected (based on the solubility parameters) plasticizer. It was assumed that a lower *Py* of the MCC could enable tablets to be prepared at lower compaction pressure and decrease the undesirable effect of compaction pressure.

## 2. Materials and Methods

### 2.1. Materials

Cellets^®^ 500 MCC cores (lot# 21E1034; IPC, Process-Center GmbH & Co KG, Grunaer Weg, Germany) were used as the starting cores. The rest of the chemicals used for the experiment, such as diethyl citrate (DEC), glycerol, and methanol were of Pharmacopeia grade and used as received.

### 2.2. Theoretical Solubility Parameter Computation

The drug–polymer miscibility was assessed theoretically via calculations of Hansen solubility parameters (HSPs) via the group contributions methodology. Thus, the energies of dispersion forces (*Ed*), polar forces (Ep), and hydrogen bonding (*Eh*) gave the dispersion (*δd*), polar (*δp*), and hydrogen bonding (*δh*) partial solubility parameters, respectively [26,27].

All calculations were performed using the Hansen Solubility Parameters in Practice (HSPiP) software (5th edition, version 5.1.03). In this study, we calculated HSPs for cellulose and DEC, while HSPs for water and glycerol were taken from the software database. It should be noted that the HSPiP database includes three sets of HSPs for water: one of them is derived from the energy of vaporisation of water at 25 °C and relates to a single molecule, whereas the other two relate to six-molecule associates which are more typical for water in a liquid state [28]. In this regard, the set of HSPs for water as associated units (based on a correlation of total miscibility with certain solvents) were used in this study.

HSPs for cellulose and DEC were calculated using the following HSPiP software DIY methods: the Yamamoto-molecular break (Y-MB), in which the components were input as simplified molecular input line entry syntax (SMILES) codes; the Van Krevelen method where the components were entered by accounting for chemical constituents and taking molar volumes from Y-MB calculations; and the Hoy method with similar input procedure as the latter one. Finally, the average HSP values within all three methods were determined.

The assessment of MCC–plasticizer miscibility was accomplished by comparing HSPs calculated according to three approaches that are based on the principle ‘like dissolves like’ [29].

The approach authored by Van Krevelen and Hoftyzer estimates a high likelihood of successful mixing of two substances if the parameter *Δδ_T_* (Equation (1)) is not more than 5 MPa^0.5^, while complete immiscibility occurs when *Δδ_T_* exceeds 10 MPa^0.5^ [30,31].

Δδ_T_ = ((δ_d1_ − δ_d2_)^2^ + (δ_p1_ − δ_p2_)^2^ + (δ_h1_ − δ_h2_)^2^)^0.5^ (1)

By Bagley’s approach, the drug–polymer miscibility is evaluated using the combined solubility parameter δv (Equation (2)).

δ_v_ = (δ_d2_ + δ_p2_)^0.5^ (2)

The probability of miscibility is concluded if the distance between two points in the two-dimensional plot is *D*_12_ ≤ 5.0 (Equation (3)) [31].

D_12_ = ((δ_v1_ − δ_v2_)^2^ + (δ_h1_ − δ_h2_)^2^)^0.5^ (3)

The approach by Greenhalgh evaluates the miscibility as the absolute difference *Δδt* (Equation (4)) between the total solubility parameters *δt* which are calculated from Equation (5).

Δδ_t_ = |δ_t1_ − δ_t2_| (4)

δ_t_ = (δ_d2_ + δ_p2_ + δ_h2_)^0.5^ (5)

According to the latter approach, drug–polymer miscibility was assumed to be likely if *Δδ_t_* ≤ 7, while *Δδ_t_* ≥ 10 MPa^0.5^ indicated immiscibility [27].

### 2.3. Plasticizer Loading onto MCC Cores Using Solvent Evaporation Method

To obtain glycerol- and DEC-loaded MCC spheres, the initial MCC spheres were dried in a vacuum oven, and their water content after drying was confirmed by Karl-Fisher (V10S; Mettler-Toledo GmbH, Greifensee, Switzerland) titration at the level of 0.1 wt.%. Two batches of plasticizer-loaded MCC spheres were made, one with DEC, using methanol as a solvent, and another with glycerol, using water as a solvent (Table 1).

About 150 g of MCC was weighed in a 500 mL round-bottom flask. Afterwards, the amount of solvent was calculated using the MCC/solvent ratio obtained from the MCC solvent absorption test. The excess solvent amount (that which could be absorbed and adsorbed by the MCC sphere) was used. The appropriate amount of plasticizer to achieve 5% loading was dissolved in the solvent. The plasticizer solution was added to MCC in a round-bottom flask (total volume of about 250 mL) and shaken vigorously by hand. The solvent was removed by a rotary evaporator (RV3 eco, from IKA-Werke GmbH & Co. KG, Staufen, Germany) at 50 °C under a pressure of 100 mbar. After that, each sample was additionally dried with dry air (50 m^3^/h) in a fluid-bed drier (Mini-Glatt; Glatt GmbH, Binzen, Germany) at 50 °C until constant outlet air temperature.

### 2.4. Thermogravimetric Analysis (TGA)

The thermal behaviour of the samples was examined using Thermal Advantage Q50 TGA (TA Instruments, New Castle, DE, USA). The samples (5–10 mg) were heated in an open aluminium pan at a heating rate of 5 °C/min or 50 °C/min from room temperature to 350 °C. Nitrogen was used as a purge gas at a flow rate of 50 mL/min for all TGA experiments. The weight remaining (%) was plotted as a function of temperature (°C). The weight loss (dM) between starting/room temperature (RT) and 200 ℃ (RT-200 °C) and temperature onset of degradation (T_d_ onset) were determined for each formulation. Data was processed with a Universal V4.5A software (TA Instruments, USA) [32].

### 2.5. Differential Scanning Calorimetry (DSC)

To investigate the thermal properties of the sample before and after processing, a heat-flux DSC (DSC Q20; TA Instruments, USA) was conducted to characterise thermal behaviour. For measurement, the samples were weighed (5–8 mg) into aluminium DSC pans and heated from −10 °C to 390 °C at 50 °C/min with a continuous purge of nitrogen gas at 50 mL/min. Melting temperature onset (Tm onset), melting peak temperature (Tm peak), and melting enthalpy were determined for each formulation. The data were processed with Universal V4.5A software (TA Instruments, USA) [10].

### 2.6. Powder X-ray Diffraction (pXRD) Analysis

The study was conducted on a diffractometer (Rigaku^TM^ Miniflex 600 C; Rigaku Co., Tokyo, Japan) in θ/2θ geometry at ambient temperature using CuKα X-radiation (λ = 1.54182 Å) at 40 kV and 15 mA power. X-ray diffraction patterns were collected over the 2θ range of 3–60° at a 5°/min scan rate. The ground sample was applied to the low-background silicone sample holder.

### 2.7. Fourier-Transform Infrared (FTIR) Attenuated Total Reflectance (ATR) Spectroscopy

FTIR-ATR study of the samples was performed on a FTIR Spectrometer (Nicolete IS20, Thermo Scientific, Karlsruhe, Germany) using a diamond prism by scanning from 4000 to 400 cm^−1^, with 2.0 cm^−1^ resolution and 100 scans per spectrum (the background was taken before each sample). Every graphically represented FTIR-profile was obtained by averaging 3 spectra.

### 2.8. Scanning Electron Microscopy (SEM) and Particle Size Distribution Analysis

SEM pictures were captured with a microscope (TM3030; Hitachi High-Tech Corp., Tokyo, Japan) in a vacuumed environment at 15 kV to obtain information about morphology on a microscopic level. The particle size distribution (D_10%_, D_50%_, and D_90%_) of the MCC spheres was determined using image analysis coupled with a VIBRI feeder and a RODOS disperser (series QICPIC/L02; Sympatec GmbH, Clausthal-Zellerfeld, Germany).

### 2.9. Preparation of Tablets

The samples (Table 2) were tableted with 11.28 mm flat punches to obtain a target mass of 500 mg using the compaction simulator STYL’One Nano (Medelpharm, Beynost, France/Korsch, Berlin, Germany). Compression cycles of a small rotary press with a turret diameter of 180 mm, a precompression roll diameter of 44 mm, an angle between rollers of 65 degrees, a compression roll diameter of 160 mm, an angle between main compression and the beginning of the compression ramp of 60 degrees, an angle of the ejection ramp of 20 degrees at a tableting speed of 70 rpm (maximum for STYL’One Nano), a precompression and compression forces of 5 and 30 kN (equivalent of 50 and 300 MPa) were used [9].

### 2.10. The Theoretical True Density Calculation

The theoretical true density of tablet composition was calculated based on the pycnometric density (*ρt*) of MCC (1.586 g/cm^3^) [16,33], glycerol (1.262 g/cm^3^) [34], DEC (1.287 g/cm^3^) [35], and their shares (*x*, *w*/*w*) using the additive methodology and the following equation [1]:

(6) $$\rho_{t}=\left(\rho_{MCC}\times x_{MCC}\right)+\left(\rho_{exc}\times x_{exc}\right)$$

### 2.11. In-Die Heckel Plot Construction

The relative density (*ln*(1/*ε*)) was calculated automatically with Alix software ver. 20220711 (Medelpharm, Beynost, France) [4]. The relative density and compaction pressure (*P*, MPa) data were plotted by the Heckel relationship [6]:

(7) $$\ln\left(1/\varepsilon \right)=K\times P+\ln\left(1/\varepsilon_{0}\right)=K\times P+A$$

where: K is the slope of the linear region (the proportionality constant), and *ln*(1/*ε*_0_) is a constant, *A*, that represents the intercept/ degree of packing (at porosity *ε*_0_) achieved at low pressure because of the rearrangement process before an appreciable amount of interparticle bonding takes place. The mean yield pressure (*P_y_*, MPa) was calculated in accordance with Hersey and Rees by the equation [3,7,36]:

(8) $$P_{y}=\frac{1}{K}$$

The mean yield pressure was measured (*n* = 10 for each formulation) in the pressure range between 70 and 210 MPa. A one-way ANOVA (analysis of variance) test was used to compare the means of two groups using the built-in possibilities of the current version of Excel (Microsoft 365; Redmond, Washington, DC, USA; Supplementary Materials).

## 3. Results and Discussion

**Figure 2:**
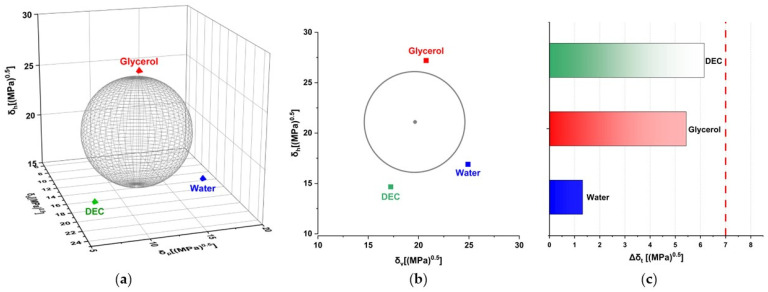
Evaluation of MCC–plasticizer miscibility using averaged solubility parameters: 3D approach authored by Hoftyzer and Van Krevelen (**a**), 2D Bagley’s plot (**b**), and 1D bar graph according to Greenhalgh (**c**).

MCC is manufactured by hydrolysis with dilute mineral acid solutions of α-cellulose sourced from raw plant material. After hydrolysis, the hydrocellulose is filtered, and the aqueous slurry is spray-dried. Thus, the MCC as an excipient contains up to 7 wt.% of moisture in accordance with pharmacopoeia (JP, PhEur, and USP) [16]. Theoretical solubility parameters were used to obtain three values (*Δδ_T_*, *D*_12_, and *Δδ_t_*) to assess the possible miscibility of cellulose with water, glycerol, and DEC (Table 3, Figure 2).

According to values averaged from the Y-MB, VK, and Hoy methods, the possible miscibility of all three plasticizers (below the proposed threshold; Table 2) was predicted only by Greenhalgh’s approach (based on Δδ_t_ calculation) which showed the following miscibility likeliness order: water > glycerol > DEC. At the same time, the other two approaches authored by Van Krevelen and Bagley, respectively, indicated that possible miscibility fell into an ambiguous region between 5 and 10 MPa^0.5^ for all studied plasticizers; however, the same likeliness order (glycerol > water > DEC) was established for both of them.

**Figure 3:**
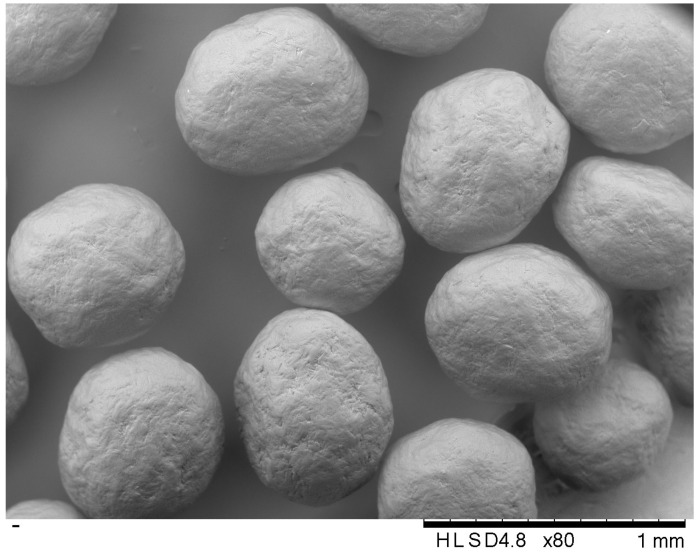
SEM of MCC spheres (D10% = 563 µm, D50% = 651 µm, and D90% = 696 µm).

**Figure 4:**
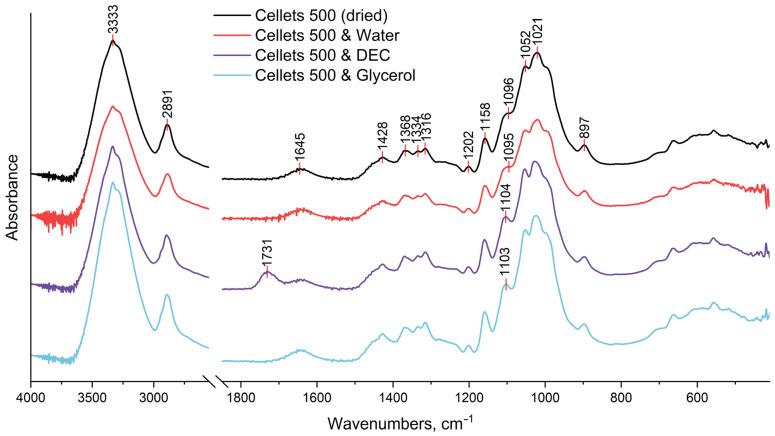
FTIR spectrum of dried and loaded MCC spheres in the range of 4000–500 cm^−1^.

Therefore, the batch of dried non-plasticized (Figure 3) and three batches of glycerol-, water-, and DEC-loaded MCC spheres were used. Plasticizer-loaded MCC spheres contained 5.0 wt.% of water, 4.2 wt.% of glycerol, and 5.2 wt.% of DEC (Table 4, Figure 4).

The dried and plasticizer-loaded MCC-spheres were investigated with FTIR spectroscopy (Figure 4). All obtained FTIR spectra showed the characteristic vibration peaks of cellulose [37,38,39,40,41,42]:

- The broad peak at 3333 cm^−1^ which is assigned to O–H stretching vibrations of the intermolecularly bonded hydroxyl group;
- The peak at 2891 cm^−1^ that corresponds to C–H stretching vibrations;
- The peak at 1645 cm^−1^ which is indicative of the O–H bending of bound water;
- The multiple absorbance bands (peaks at 1428, 1368, 1334, and 1316 cm^−1^) assigned to the bending and stretching vibrations of C–H and C–O bonds;
- The peaks at 1202, 1052, and 1021 cm^−1^ are assigned to the elongation of C-O bonds;
- The peaks at 1158 and 897 cm^−1^ are due to the C–O–C stretching vibrations at the β-glycosidic linkage.

No evident differences were observed in the spectrum of water-plasticized MCC spheres compared to the dried non-plasticized sample. This could be explained by the remaining bound water in all samples even after drying (as evidenced by the persistence of the peak at 1645 cm^−1^ in all obtained spectra [37,41,42]. Nonetheless, some changes were established for MCC spheres treated with DEC and glycerol. Both these plasticizers led to the manifestation of the peak at ~1104 cm^−1^, which could be related to the stretching vibrations of the C–O bond in the ester group of DEC and the secondary alcohol group of glycerol [43,44,45]. In addition, the spectrum of DEC-loaded MCC spheres demonstrated the most explicit deviation from that of the dried MCC spheres that manifested as a peak at 1731 cm^−1^ which was absent in the spectra of all other three samples. This peak could be assigned to the C=O stretching of the ester functional group [43]. Therefore, it can be suggested that treatment of MCC spheres with DEC and glycerol resulted in intermolecular hydrogen bonding between hydroxyl groups of cellulose (hydrogen donor) and mentioned functional groups of these plasticizers (hydrogen acceptors), and thus, at the molecular level, the plasticization could be caused by a weakening of intermolecular hydrogen bonds between adjacent cellulose chains [46]. It is interesting to note that it was the secondary alcohol hydroxy group of glycerol (at 1103 cm^−1^), and not the primary ones (at ~1030 cm^−1^) [45], that appeared in the spectrum of the glycerol-loaded MCC. As a rule, glycerol primary hydroxy groups are more reactive, and because of that, they are more likely to be involved in homo-intermolecular hydrogen bonding (i.e., glycerol–glycerol). With loading into MCC spheres, hetero-intermolecular hydrogen bonding occurred, i.e., cellulose–glycerol, which apparently was mostly contributed by the secondary alcohol hydroxy group of glycerol, while the homo-glycerol hydrogen bonding network could be preserved. Analogue findings were demonstrated in the study of the glycerol–choline eutectic mixture, which was found to have homo-molecular glycerol hydrogen bonding network similar to that in pure glycerol, whereas choline bonds were at the interstitial voids of the glycerol network [47].

**Figure 5:**
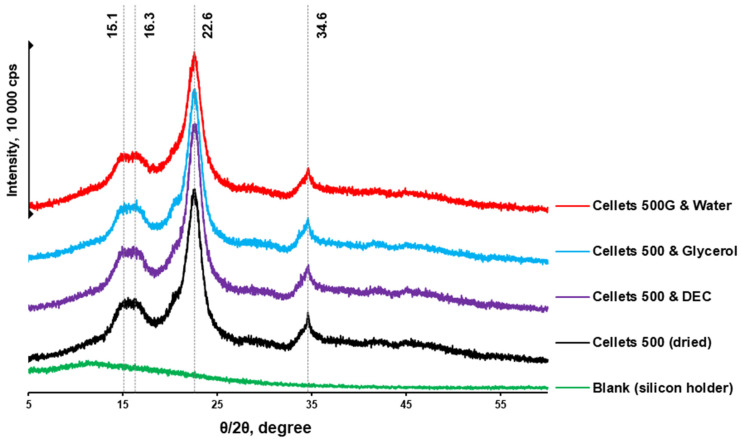
pXRD diffractograms of dried and loaded MCC spheres.

pXRD is a complementary technique to DSC and was used in assessing the presence of crystalline content in formulations. Thus, the pXRD profiles of dried and plasticizer-loaded MCC spheres were investigated. The diffraction patterns of all samples confirmed the crystalline nature of each sample with the same characteristic peaks (Figure 5). The characteristic MCC peaks were also shown to be similar to that reported in the literature [48]. Unfortunately, the pXRD method was reported to have relatively low sensitivity and a limit of detectability (LoD) of 5% [49,50]. Thus, considering the plasticizer load (approx. 5%), the pXRD profiles obtained can be considered similar (with approximately the same level of crystallinity).

**Figure 6:**
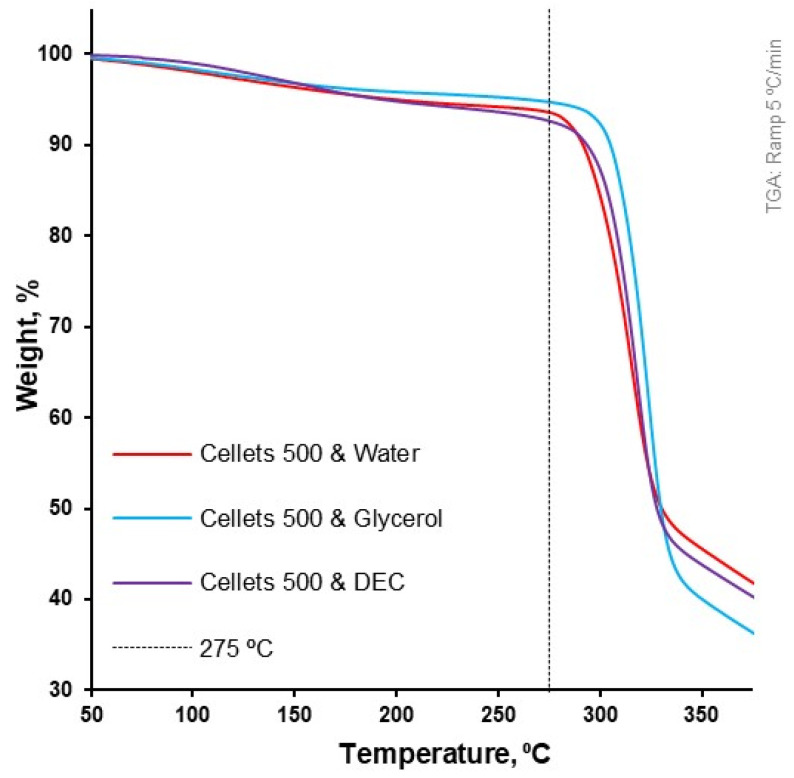
Weight loss as a function of temperature for loaded MCC spheres (TGA at 5 °C/min).

At a 5 °C/min heating rate, the onset of degradation temperature (Td onset) increased from water to DEC and glycerol (from 297.4 to 303.2 and 309.2 °C, respectively; Table 4, Figure 3). Melting of the MCC (DSC-curves) was observed upon its degradation (TGA-curve; Table 4, Figure 4). The increase in heating rate up to 50 °C/min made it possible to increase the Td onset for water-loaded MCC up to 345.7 °C and compare the melting onset temperatures (Tm onset) for MCC loaded with plasticizers. The part of the DSC curve that described melting demonstrated a two-step shape and was characterized by two Tm onsets. The increase in Tm onset 1 and Tm onset 2 was in the same sequence and increased from water to DEC and glycerol: 291.9, 305.7, 315.8 °C for Tm onset 1 and 325.6, 332.4, 335.6 °C for Tm onset 2, respectively (Table 4, Figure 6).

**Figure 7:**
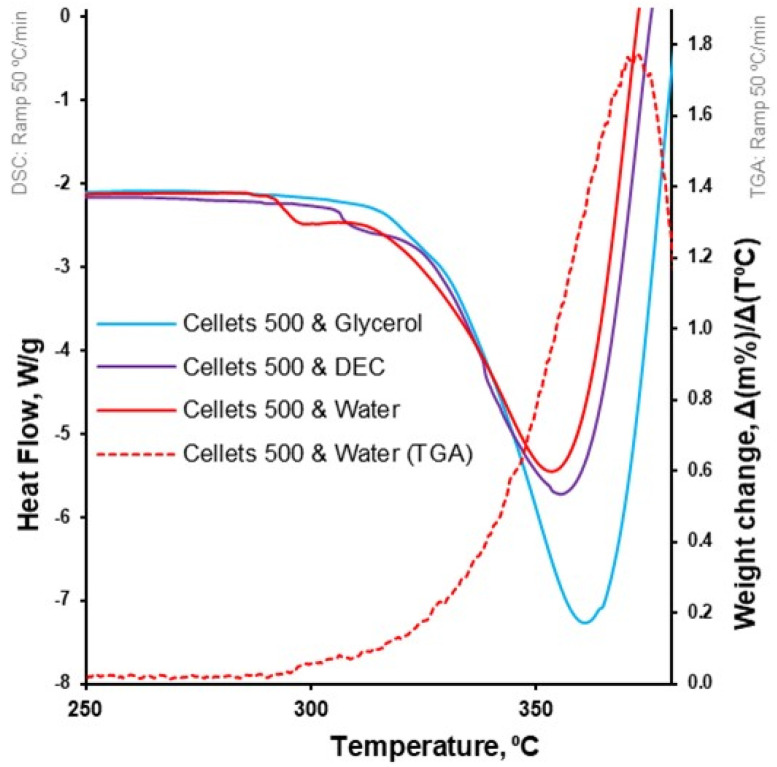
Heat flow as function of temperature for loaded MCC spheres (DSC at 50 °C/min; left Y-axis); the first derivative of weight loss as a function of temperature for water-loaded MCC spheres (TGA at 50 °C/min; right Y-axis).

In this study, the T_m_ onset 1 and 2 (for water- and DEC-loaded samples) was associated with the thermal degradation of MCC [51]. That can be observed by comparing the first derivative of weight loss and respective Tm onset on the DSC profile of water-loaded MCC spheres (Figure 7). The increase in apparent melting peak temperature (Tm) and apparent melting enthalpy can be explained with the increase in T_d_ from water to DEC and glycerol. Therefore, the thermal analysis did not provide us with insights regarding the plasticization of MCC with selected plasticizers.

**Figure 8:**
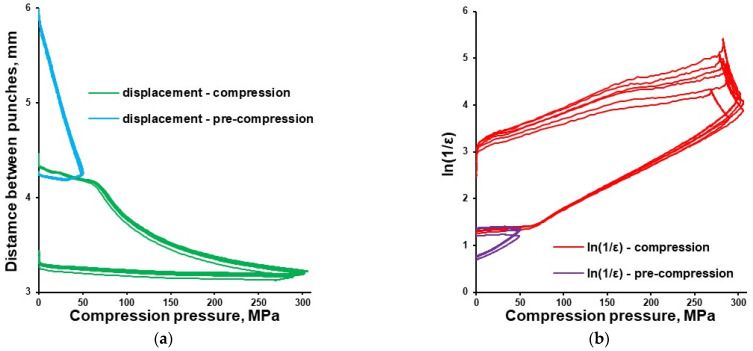
In-die Heckel plot (**a**) and pressure-displacement profile (**b**) for MCC spheres (Cellets^®^ 500) loaded with glycerol.

Tableting of plasticizer-loaded MCC spheres with a compaction simulator was illustrated with pressure-displacement profiles (Figure 8a; exemplified with glycerol-loaded MCC spheres), which were converted to in-die Heckel plots (Figure 8b).

**Figure 9:**
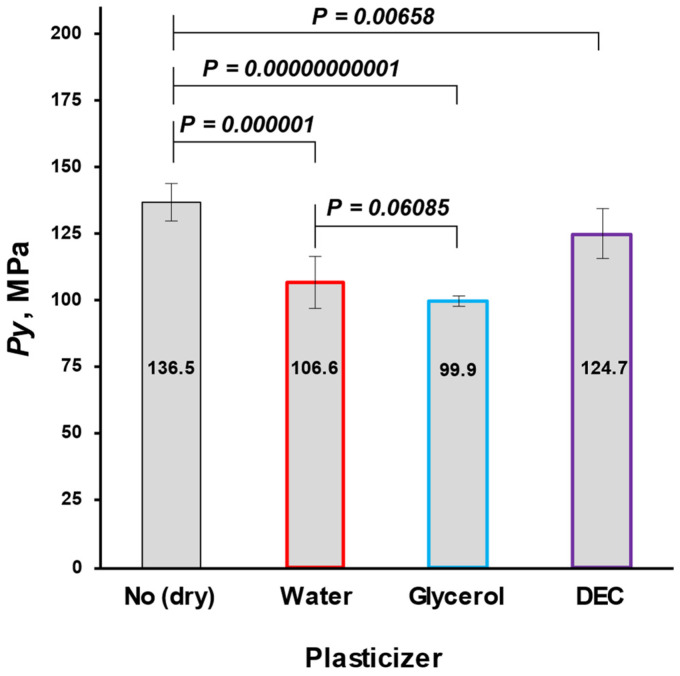
Comparison of non-plasticized (dried) with plasticized MCC spheres (Cellets^®^ 500) in terms of *Py* (a plasticity parameter).

The mean yield pressure (*Py*) of non-plasticized MCC was found at the level of 136.5 ± 6.9 MPa (Av. ± S.D.). Despite the sequence of Tm onsets, the mean yield pressure of plasticizer-loaded MCC spheres decreased from DEC (124.7 ± 9.2 MPa) to water (106.6 ± 10.0 MPa) and glycerol (99.9 ± 1.9 MPa; Figure 9, Table 5). That coincided with the miscibility likelihood order based on the HSP calculations. Therefore DEC, water, and glycerol were able to decrease the *Py* of non-plasticized MCC spheres by 4.7, 16.3, and 38.9%, respectively.

Interestingly, despite FTIR revealing more hydrogen binding sites in the case of treatment with DEC (i.e., both C–O and C=O bonds of the ester functional group), glycerol with only one binding site (C–O bond of the alcohol group) was superior in its plasticizing ability, implying that MCC–glycerol hydrogen bonding was more efficient. This could be explained from the viewpoint of molecular weights of glycerol and DEC (92.09 and 248.23 g/mol, respectively). Considering an equal mass loading of both plasticizers (7.88 g), the loading of glycerol was 2.5 times higher in terms of molarity; therefore, more molecules of plasticizer were involved and, accordingly, more hydrogen bonds with cellulose could be formed in the case of glycerol. This follows the general logic that the smaller the molecule weight, the greater the plasticization effect of the plasticizer upon the polymer matrix [52]; however, the strength of intermolecular interactions should also be considered.

Water, as an MCC plasticizer, showed a relatively high ability to decrease *Py* (increase plasticity). The results obtained highlight the importance of water content in the raw MCC material. Changing the MCC plasticity by 16.3% (at 5 wt.%) significantly changed the mechanical properties. Thus, the fluctuation of moisture content in the MCC (even in the eligible pharmacopeial range) can be the reason for the variability of mechanical properties in complex tablet formulations [53]. Considering moisture as one of the most important factors in pharmaceutical tablets’ shelf-life, narrow specification of moisture content in MCC during the product development stage can be recommended.

## 4. Conclusions

This study showed the possibility of increasing the plasticity of MCC by loading it with a deliberately chosen plasticizer. The computational approaches based on solubility parameters were found to be useful in predicting the plasticizing efficacy. Based on FTIR findings, it is suggested that plasticization resulted from intermolecular hydrogen bonding between the plasticizers and cellulose molecules that caused the weakening of hydrogen bonds between adjacent cellulose chains. At the plasticizer load used (approx. 5 wt.%), neither pXRD nor DSC gave any insights on the plasticization of MCC with selected plasticizers. Because of the relatively high plasticization ability of water towards cellulose and thus potential changes in MCC mechanical properties, narrow specification of moisture content in MCC during the product development stage can be recommended.

## Figures and Tables

**Table 1:** Used amounts of plasticizer and solvent for the plasticizer loading procedure.

MCC	Plasticizer		Solvent			
Mass, g	Type	Mass, g	Type	g/mL	Mass, g	mL *
150	DEC	7.88	Methanol	0.792	34.5	44
150	Glycerol	7.88	Water	0.997	93.0	93

* The volume was calculated from the mass using the density.

**Table 2:** Formulations for tableting.

	Cellets 500 and Water	Cellets 500 and DEC	Cellets 500 and Glycerol
Ingredients	*w*/*w*	*w*/*w*	*w*/*w*
MCC	95.0	94.8	95.8
Moisture	5.0	–	–
DEC	–	5.2	–
Glycerol	–	–	4.2

**Table 3:** Hansen solubility parameter calculations.

Calculation Method	Δδ_T_ (Highly Likely Miscible If ≤$5\sqrt{MPa}$)			D_12_ (Highly Likely Miscible If ≤$5.0\sqrt{MPa}$)			Δδ_t_ (Highly Likely Miscible If ≤$7\sqrt{MPa}$		
	Water	Glycerol	DEC	Water	Glycerol	DEC	Water	Glycerol	DEC
Y-MB	6.74	9.14	5.20	4.73	9.11	4.41	2.88	7.00	3.87
VK	13.29	6.31	9.63	11.28	5.49	9.38	1.05	5.16	6.15
Hoy	5.36	9.40	9.47	4.47	6.64	9.06	1.39	2.73	9.05
Averaged	7.29	6.33	7.53	6.79	6.21	6.84	1.32	5.43	6.15

**Table 4:** The summary of thermal properties determined by TGA and DSC.

		dM (RT-200 °C)	Td Onset	Td Onset	Tm Onset 1	Tm Onset 2	Tm Peak	Melting Enthalpy
		% (*w*/*w*)	°C	°C	°C	°C	°C	J/g
Cellets 500 and Water	Av.	5.0	297.4	345.7	291.9	325.6	353.4	134.2
	S.D.	0.2	–	–	1.4	1.4	0.1	4.2
Cellets 500 and DEC	Av.	5.2	303.2	–	305.7	332.4	356.7	131.6
	S.D.	0.3	–	–	1.1	0.7	1.4	7.6
Cellets 500 and Glycerol	Av.	4.2	309.3	–	315.8	335.6	360.5	194.3
	S.D.	0.3	–	–	0.8	0.6	0.3	2.5
**Method used**		TGA	TGA	TGA	DSC	DSC	DSC	DSC
**Heating rate**		5 °C/min	5 °C/min	50 °C/min	50 °C/min	50 °C/min	50 °C/min	50 °C/min

**Table 5:** *Py* data statistics.

	Plasticizer			
	No (Dried)	Water	Glycerol	DEC
n	10	10	10	10
min	124.8	92.6	97.1	113.6
max	145.7	122.0	102.0	138.9
Av.	136.5	106.6	99.9	124.7
S.D.	6.9	10.0	1.9	9.2

## Data Availability

The data presented in this study are available on request from the corresponding author.

## Supplementary Materials

The following supporting information can be downloaded at: https://www.mdpi.com/article/10.3390/pharmaceutics16070945/s1, Table S1. Raw values of *Py*; Statistical analysis: One-Way ANOVA (α = 0.05).
