# Supplementary material for: The Increase in the Plasticity of Microcrystalline Cellulose Spheres’ When Loaded with a Plasticizer
Source: Pharmaceutics. 2024 Jul 16;16(7):945. doi: 10.3390/pharmaceutics16070945 (PMC11279479; doi:10.3390/pharmaceutics16070945)
Supplement: Supplementary file 1 [file pharmaceutics-16-00945-s001.zip › pharmaceutics-3069456-supplementary.pdf]

pharmaceutics

MDPI

Table S. Raw values of *Py*

	Plasticizer			
#	**No (dry)**	**Water**	**Glycerol**	**DEC**
1	140.0	92.6	97.1	113.6
2	129.5	93.5	97.1	114.9
3	135.5	98.0	100.0	116.3
4	127.1	100.0	100.0	119.0
5	140.0	106.4	100.0	120.5
6	144.2	106.4	102.0	123.5
7	141.6	112.4	102.0	126.6
8	145.7	116.3	97.5	135.1
9	124.8	119.0	101.1	138.9
10	136.8	122.0	101.7	138.9

Statistical analysis: One-Way ANOVA (α = 0.05)

	Groups	*Count*	*Sum*	*Average*	*Variance*		
SUMMARY	**No (dry)**	10	1365.2	136.52	52.19733		
	**Water**	10	1066.493	106.6493	111.4776		
	Source of Variation	*SS*	*df*	*MS*	*F*	*P-value*	*F crit*
ANOVA	Between Groups	4461.289	1	4461.289	54.51403	**7.5E-07**	4.4139
	Within Groups	1473.074	18	81.83744			
	Total	5934.363	19				

	Groups	*Count*	*Sum*	*Average*	*Variance*		
SUMMARY	**No (dry)**	10	1365.2	136.52	52.19733		
	**Glycerol**	10	998.5564	99.85564	3.937576		
	Source of Variation	*SS*	*df*	*MS*	*F*	*P-value*	*F crit*
ANOVA	Between Groups	6721.377	1	6721.377	239.4723	**7.6E-12**	4.4139
	Within Groups	505.2142	18	28.06745			
	Total	7226.591	19				

	Groups	*Count*	*Sum*	*Average*	*Variance*		
SUMMARY	**No (dry)**	10	1365.2	136.52	52.19733		
	**DEC**	10	1247.339	124.7339	94.98712		
	Source of Variation	*SS*	*df*	*MS*	*F*	*P-value*	*F crit*
ANOVA	Between Groups	694.555	1	694.555	9.437885	**6.6E-03**	4.4139
	Within Groups	1324.66	18	73.59223			
	Total	2019.215	19				

	Groups	*Count*	*Sum*	*Average*	*Variance*		
SUMMARY	**Water**	10	1066.493	106.6493	111.4776		
	**Glycerol**	10	998.5564	99.85564	3.937576		
	Source of Variation	*SS*	*df*	*MS*	*F*	*P-value*	*F crit*
ANOVA	Between Groups	230.7703	1	230.7703	3.998961	**6.1E-02**	4.4139
	Within Groups	1038.736	18	57.70756			
	Total	1269.506	19				
